# Evolution of Ciprofloxacin Resistance-Encoding Genetic Elements in *Salmonella*

**DOI:** 10.1128/mSystems.01234-20

**Published:** 2020-12-22

**Authors:** Kaichao Chen, Chen Yang, Ning Dong, Miaomiao Xie, Lianwei Ye, Edward Wai Chi Chan, Sheng Chen

**Affiliations:** a Department of Infectious Diseases and Public Health, Jockey Club College of Veterinary Medicine and Life Sciences, City University of Hong Kong, Kowloon, Hong Kong; b State Key Laboratory of Chemical Biology and Drug Discovery, Department of Applied Biology and Chemical Technology, The Hong Kong Polytechnic University, Hung Hom, China; Marquette University

**Keywords:** *Salmonella*, ciprofloxacin resistance, plasmids, PMQR genes, phylogenetic analysis, evolution

## Abstract

The incidence of ciprofloxacin resistance in *Salmonella* has increased dramatically in the past decade. To track the evolutionary trend of ciprofloxacin resistance-encoding genetic elements during this period, we surveyed the prevalence of *Salmonella* in food products in Shenzhen, China, during the period of 2012 to 2017 and performed whole-genome sequencing and genetic analysis of 566 ciprofloxacin-resistant clinical *Salmonella* strains collected during this survey. We observed that target gene mutations have become much less common, with single *gyrA* mutation currently detectable in Salmonella enterica serovar Typhimurium only. Multiple plasmid-mediated quinolone resistance (PMQR) genes located in the chromosome and plasmids are now frequently detectable in ciprofloxacin-resistant *Salmonella* strains of various serotypes. Among them, the *qnrS1* gene was often harbored by multiple plasmids, with p10k-like plasmids being the most dominant. Importantly, p10k-like plasmids initially were not conjugative but became transmissible with the help of a helper plasmid. Ciprofloxacin resistance due to combined effect of carriage of the *qnrS1* gene and other resistance mechanisms is common. In *S*. Typhimurium, carriage of *qnrS1* is often associated with a single *gyrA* mutation; in other serotypes, combination of *qnrS1* and other PMQR genes located in the chromosomal fragment or plasmid is observed. Another major mechanism of ciprofloxacin resistance, mainly observable in *S*. Derby, involves a chromosomal fragment harboring the *qnrS2–aac(6′)lb-cr–oqxAB* elements. Intriguingly, this chromosomal fragment, flanked by IS*26*, could form a circular intermediate and became transferrable. To conclude, the increase in the incidence of various PMQR mobile genetic elements and their interactions with other resistance mechanism contribute to a sharp increase in the prevalence of ciprofloxacin-resistant clinical *Salmonella* strains in recent years.

**IMPORTANCE** Resistance of nontyphoidal *Salmonella* to fluoroquinolones such as ciprofloxacin is known to be mediated by target mutations. This study surveyed the prevalence of *Salmonella* strains recovered from 2,989 food products in Shenzhen, China, during the period 2012 to 2017 and characterized the genetic features of several PMQR gene-bearing plasmids and ciprofloxacin resistance-encoding DNA fragments. The emergence of such genetic elements has caused a shift in the genetic location of ciprofloxacin resistance determinants from the chromosomal mutations to various mobile genetic elements. The distribution of these PMQR plasmids showed that they exhibited high serotype specificity, except for the p10k-like plasmids, which can be widely detected and efficiently transmitted among *Salmonella* strains of various serotypes by fusing to a new conjugative helper plasmid. The sharp increase in the prevalence of ciprofloxacin resistance in recent years may cause a predisposition to the emergence of multidrug-resistant *Salmonella* strains and pose huge challenges to public health and infection control efforts.

## Abstract (precis)

Resistance of nontyphoidal *Salmonella* to fluoroquinolones such as ciprofloxacin is known to be mediated by target mutations. This study surveyed the prevalence of *Salmonella* strains recovered from 2,989 food products in Shenzhen, China, during the period 2012 to 2017 and characterized the genetic features of several PMQR gene-bearing plasmids and ciprofloxacin resistance-encoding DNA fragments.

## INTRODUCTION

*Salmonella* spp. remain one of the most important bacterial pathogens that cause foodborne diseases. Although the majority of *Salmonella* infections are self-limiting, they may occasionally cause systemic infections, for which the mortality rate is particularly high among immunocompromised and elderly patients. In such cases, antimicrobial treatment is required (1, 2). Three antibiotics have been approved by the FDA in the United States to treat infections caused by *Salmonella*: ciprofloxacin, ceftriaxone, and azithromycin (3–6). In recent years, however, resistance to these antibiotics has been increasingly common, with a particularly high rate being reported in Asia. Importantly, this rapid increase in the rate of resistance to ciprofloxacin is observable among all serotypes of *Salmonella*.

In the past, mutational changes in target genes, which often involved double mutations in the *gyrA* gene and a single mutation in the *parC* gene, were the primary mechanisms of resistance to ciprofloxacin (7, 8). Since 2000, plasmid-mediated quinolone resistance (PMQR) determinants, such as *qnrA*, *qnrB*, *qnrC*, *qnrD*, *qnrS*, *aac(6′)lb-cr*, and *oqxAB*, are being increasingly reported in *Salmonella* (9–14). PMQR genes normally mediate expression of quinolone resistance and reduction in susceptibility to ciprofloxacin. However, single mutation in *gyrA*, along with carriage of a PMQR gene, was found to mediate expression of ciprofloxacin resistance in *Salmonella* (15). In addition, ciprofloxacin resistance mediated by multiple PMQR genes is not uncommon (16–18). Nevertheless, these new mechanisms normally mediate a lower level of resistance to ciprofloxacin, with a MIC of >8 μg/ml being commonly reported in strains of various serotypes (14–16, 18). However, one feature of these new resistance mechanisms is that they are encoded by mobile genetic elements, thereby enabling *Salmonella* to acquire ciprofloxacin resistance rapidly without having to pay the fitness cost associated with mutational changes in the drug target genes. Most of the currently known PMQR-encoding plasmids are nonconjugative, limiting the rate by which they are transmitted among *Salmonella* strains. However, several studies have recently reported the discovery of conjugative plasmids that harbor PMQR genes in *Salmonella* and other Gram-negative bacteria (18). Worse still, conjugative helper plasmids that can be fused with nonconjugative PMQR-encoding plasmids and mediate their transmission among *Salmonella* strains have also been reported (17). However, these studies did not show how genetic elements that encode ciprofloxacin resistance evolved in *Salmonella*. In this work, we conducted surveillance of *Salmonella* strains recovered in various food samples in Shenzhen, China, during the period 2012 to 2017 and witnessed a rapid increase in the incidence of ciprofloxacin resistance in foodborne *Salmonella* isolates. Whole-genome sequencing and genetic analysis enable us to depict the molecular mechanisms underlying the rapid evolution of ciprofloxacin resistance-encoding genetic elements in different serotypes of *Salmonella* and provide valuable insights into the key genetic elements concerned, facilitating the development of new strategies to control the dissemination of resistance-encoding elements in *Salmonella*.

## RESULTS

### Prevalence of *Salmonella* isolates in food products.

A total of 1,116 nonrepeated *Salmonella* strains were collected from 2,989 food samples purchased during the period of 2012 to 2017 in Shenzhen, China, as part of the food safety surveillance program organized by the Shenzhen Government. Among the 2,989 food samples, 1,558 were purchased from wet markets and 1,131 were purchased from supermarkets. These food samples contained 1,459 pork, 230 beef, 543 chicken, and 342 shrimp samples. Among the 1,116 *Salmonella* isolates, 82 (26%) were isolated from 317 food samples in 2013, 157 (25%) from 440 food samples in 2014, 287 (23%) from 754 food samples in 2015, 445 (23%) from 1,107 food samples in 2016, and 145 (20%) from 371 food samples in 2017 (Table 1). Among these *Salmonella* isolates, 746 were isolated from the 1,459 pork samples (30%), 282 were recovered from the 543 chicken samples (28%), 65 were from the 230 beef samples (17%), and 22 were from the 342 shrimp samples (5%) (Table 1).

**TABLE 1:** Prevalence of *Salmonella* in different food products in Shenzhen, 2013 to 2017 (*n* = 2,689)

Yr	No. of food samples	No. of *Salmonella* strains in different food samples and isolation rates (%)													
		No. of isolates	Isolation rate (%)	Pork		Chicken		Beef		Shrimp		Cip^r^ isolates		CRO^r^ isolates^*a*^	
				*n*	%	*n*	%	*n*	%	*n*	%	*n*	%	*n*	%
2013	317	82	26	52	26	25	23					30	37	14	17
2014	440	157	26	134	28	23	25					61	39	12	8
2015	754	287	23	192	28	70	28	16	14	8	5	138	48	8	3
2016	1,107	445	24	264	24	127	36	47	26	14	6	226	51	70	16
2017	371	145	20	104	30	37	16	2	2	0	0	112	77	36	25
Total	2,989	1,116	24	746	30	282	28	65	17	22	4	566	51	140	13

a CRO, ceftriaxone.

### Antimicrobial susceptibility of *Salmonella* food isolates.

These *Salmonella* isolates were found to be resistant to most of the antibiotics tested, with 51%, 13%, and 5% being resistant to ciprofloxacin, ceftriaxone, and azithromycin, respectively. These three antibiotics are the current choices for treatment for *Salmonella* infections. The rate of resistance to other agents is 76% to tetracycline, 60% to sulfamethoxazole-trimethoprim, 60% to ampicillin, 52% to chloramphenicol, and 42% to nalidixic acid. On the other hand, these strains were mostly susceptible to meropenem (100%), amikacin (97%), and kanamycin (75%). It is worrisome that a much higher proportion of *Salmonella* isolates that were resistant to ciprofloxacin, ceftriaxone, and azithromycin were also resistant to other antibiotics, unlike isolates that were susceptible to these three antibiotics. The rate of resistance to ciprofloxacin was also found to have increased significantly over the years, from 39% (33/82) in 2012 to 2013 to 77% (112/145) in 2017. Cefotaxime resistance rate, on the other hand, decreased significantly, from 17% (14/82) in 2012 to 2013 to 8% (12/157) in 2014 and then to 3% (8/290) in 2015, yet the rate increased again and reached 16% (70/445) in 2016 and further climbed to 25% (36/145) in 2017 (see Table S1 in the supplemental material).

10.1128/mSystems.01234-20.1 TABLE S1 Resistance rate, MIC_50_, and MIC_90_ of foodborne *Salmonella* isolates. Download Table S1, DOCX file, 0.02 MB. Copyright © 2020 Chen et al. 2020 Chen et al. This content is distributed under the terms of the Creative Commons Attribution 4.0 International license.

### Ciprofloxacin resistance in different serotypes of *Salmonella*.

The 1,116 *Salmonella* isolates were found to belong to 54 serotypes, of which Salmonella enterica serovar Derby, *Salmonella* Typhimurium, and *Salmonella* Rissen were the most common, accounting for 24%, 17%, and 9% of all test strains, respectively. Each of several serotypes of *Salmonella* isolates, such as *Salmonella* Corvallis, *Salmonella* London, and *Salmonella* Agona, accounted for approximately 6% of the test strains. These serotypes of *Salmonella* were also prevalent among those that were resistant to ciprofloxacin. Although the distribution patterns of these serotypes, including *Salmonella* Corvallis strains, which were recovered after 2015, were highly similar over the past few years, the ciprofloxacin resistance rate of these serotypes has increased sharply, reaching the highest rate in the year 2017. Among the 269 *S*. Derby isolates tested, resistance to ciprofloxacin increased dramatically over the years, with a rate of 40%, 32%, 54%, 50%, and 86% being recorded in years 2012 to 2013, 2014, 2015, 2016, and 2017, respectively (Table 2). Likewise, ciprofloxacin-resistant strains accounted for 64%, 59%, 57%, 48%, and 84% of the *Salmonella* Typhimurium isolates collected in these 5 years (a total of 198 isolates), respectively, suggesting that the high resistance rate recorded throughout the study period increased further in 2017. For the 104 *Salmonella* strains that belonged to the serotype Rissen, none of the isolates recovered in 2012 to 2013 and 2014 was resistant to ciprofloxacin, yet 7%, 29%, and 57% of isolates were found to be ciprofloxacin resistant in 2015, 2016, and 2017, respectively. A similar trend of ciprofloxacin resistance was observed in other serotypes of *Salmonella*, with some exhibiting over 90% resistance (*S*. Corvallis, S. London, *S*. Kentucky, and *S*. Give) (Table 2).

**TABLE 2:** Prevalence rate and mechanism of ciprofloxacin resistance in various *Salmonella* serotypes

No.	Serotype	No. of isolates/no. of Cip^r^ isolates (%)						No. of target mutations		PMQR mobile element^*a*^			
		2012–2013	2014	2015	2016	2017	Total	*gyrA*	*parC*	1	2	3	4
1	*S*. Derby	32/13 (41)	52/17 (33)	74/40 (54)	81/41 (51)	30/26 (87)	269/137 (51)	2	0	+	+	+	+
2	*S*. Typhimurium	17/11 (65)	13 (59)	38/22 (58)	89/43 (48)	32/27 (84)	198/116 (59)	71	2	+	+	+	+
3	*S*. Rissen	5/0 (0)	20/0 (0)	41/3 (7)	24/7 (29)	14/8 (57)	104/18 (17)	2	0	+	−	−	−
4	*S*. Corvallis	0/0 (0)	0/0 (0)	19/19 (100)	39/35 (90)	9/8 (89)	67/62 (93)	0	0	+	+	+	−
5	*S*. Agona	1/0 (0)	3/2 (67)	22/19 (86)	37/30 (81)	6/6 (100)	69/57 (83)	1	0	+	−	−	−
6	*S*. London	4/2 (50)	20/8 (40)	10/8 (80)	22/11 (50)	9/8 (89)	65/37 (57)	0	0	+	−	+	+
7	*S*. Mbandaka	1/1 (100)	0/0 (0)	12/8 (67)	21/6 (29)	0/0 (0)	33/15 (46)	1	0	+	−	−	−
8	*S*. Stanley	0/0 (0)	5/0 (0)	12/0 (0)	7/2 (29)	1/1 (100)	25/3 (12)	1	0	+	−	−	−
9	*S*. Kentucky	0/0 (0)	0/0 (0)	6/6 (100)	17/16 (94)	15/15 (100)	38/37 (97)	4	4	+	−	+	−
10	*S*. Enteritidis	16/1 (6)	0/0 (0)	2/0 (0)	2/0 (0)	4/1 (25)	24/2 (8)	16	0	+	−	−	−
11	*S*. Meleagridis	0/0 (0)	9/3 (33)	2/0 (0)	7/4 (57)	3/2 (67)	21/9 (43)	1	0	+	−	+	+
12	*S*. Give	0/0 (0)	0/0 (0)	0/0 (0)	5/5 (100)	0/0 (0)	5/5 (100)	5	0	+	−	−	−

a PMQR mobile elements: 1, *qnrS1* carrying; 2, *qnrS2–aac(6′)lb-cr–oqxAB*; 3, *qnrB6-qnrB4–aac(6′)lb-cr*; 4, *gryA* single mutation combined with *aac(6′)lb-cr–oqxAB*.

### Evolution of genetic elements that encode ciprofloxacin resistance in *Salmonella* isolates.

**FIG 1:**
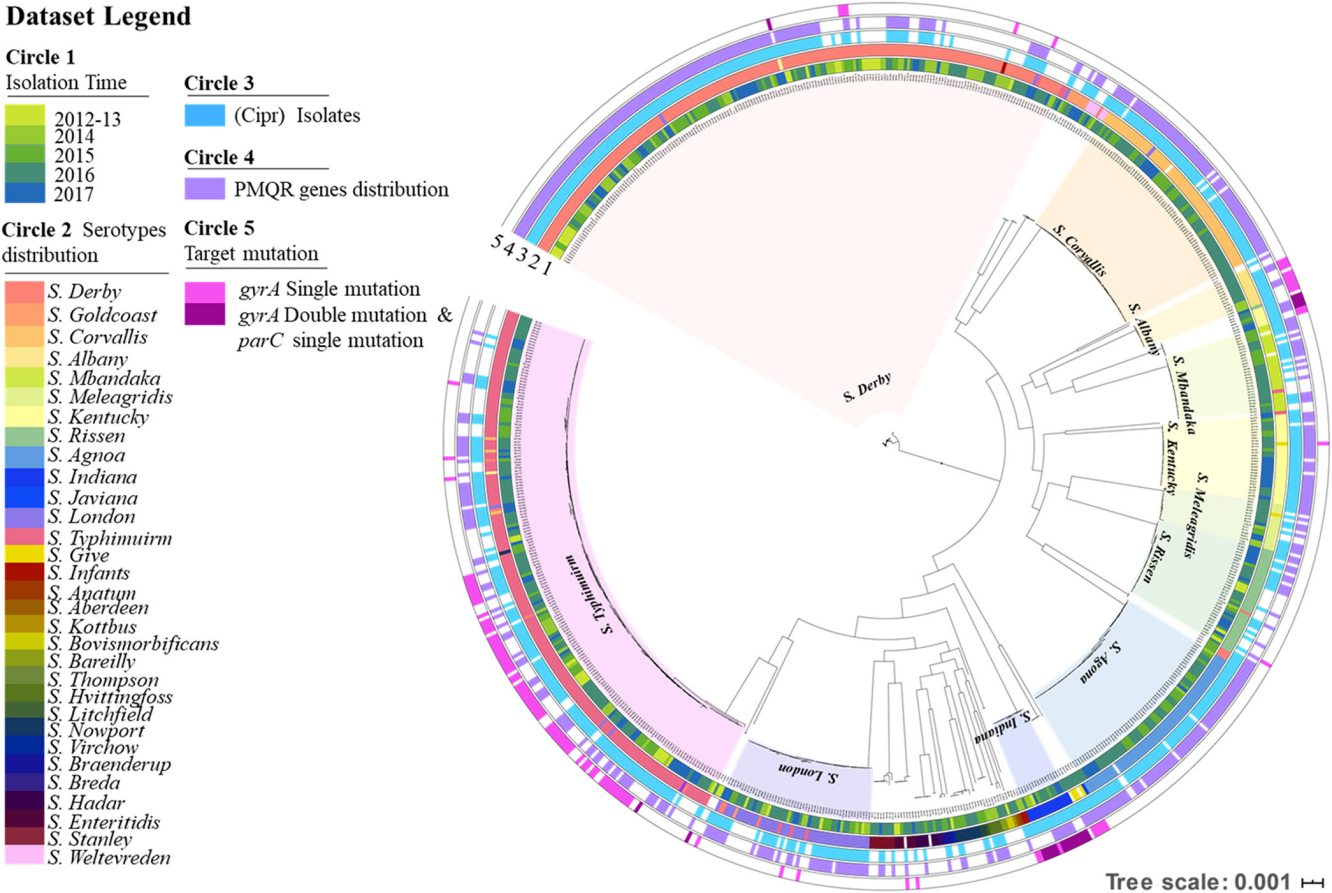
Phylogenetic tree of different serotypes and sequence types among 816 *Salmonella* isolates collected during the period of 2012 to 2017. Circle 1 depicts strain isolation time; circle 2 denotes distribution of serotypes, using various colors shown in the data set legend (the main serotypes are also highlighted in clades of different colors); circle 3, of blue color, denotes ciprofloxacin-resistant isolates; and circle 4 and circle 5, of purple and deep red color, denote the distribution of PMQR genes and target mutations, respectively.

To further investigate the genetic basis of variation in the prevalence of ciprofloxacin resistance in *Salmonella* during the study period, phylogenetic and genomic analyses of 816 isolates, including 250 ciprofloxacin-sensitive (Cip^s^) and 566 ciprofloxacin-resistant (Cip^r^) isolates, were performed to study the pattern of distribution of PMQR genes and the types of target mutations among the resistant strains as well as the mechanism of transfer of resistance-encoding genetic elements between resistant and susceptible *Salmonella* strains. Ciprofloxacin resistance was serotype dependent, with some serotypes exhibiting a very high rate, whereas a low rate was recorded in the others. No significant genetic differences were observed between Cip^r^ and Cip^s^ *Salmonella* strains within the same serotype (Fig. 1). Ciprofloxacin resistance was mainly mediated by PMQR genes, with 95% (539/566) of Cip^r^ isolates being found to harbor PMQR genes; importantly, 78% (445/566) of these PMQR gene-bearing Cip^r^ strains did not harbor any mutations in the quinolone resistance-determining regions (QRDR) of the target genes. The carriage rate of PMQR genes in *Salmonella* isolates increased from 20% in 2012 to 2013 to 65% in 2017. These genes were commonly detected in serotypes such as *S*. Derby, *S*. London, *S*. Typhimurium, *S*. Risen, *S*. Corvallis, and *S*. Agona (Fig. 1). Target mutations were detected in 122 of the 566 Cip^r^ isolates and were most commonly detected in strains of the following serotypes: *S*. Typhimurium (74/122), *S*. Infantis (3/122), *S*. Kentucky (7/122). *S*. Albany (9/122), and *S*. Indiana (15/122). However, the rate of carriage of target gene mutations among the Cip^r^ *Salmonella* strains was found to decline steadily from 39% (13/33) in 2013 to 15% (17/145) in 2017, despite a marked increase in the prevalence of ciprofloxacin-resistant strains during this period. Among the Cip^r^ strains with target mutations, *S*. Typhimurium, *S*. Infantis, and *S*. Albany contained only a single mutation in the *gyrA* gene (S^83^F, S^83^G, S^83^L, S^83^N, S^83^Y, D^87^N, D^87^Y, or D^87^G), whereas *S*. Indiana and *S*. Kentucky harbored the double mutation S^83^L and D^87^Y in the *gyrA* gene and a single mutation, S^80^I/S^80^R, in the *parC* gene. Importantly, different PMQR genes, including *qnrB6-qnrB4*, *qnrS1*, *oqxAB*, and *aac(6′)Ib-cr*, were concurrently detected in isolates that contained target gene mutations, especially isolates collected after 2014. Overall, among the 566 Cip^r^ *Salmonella* isolates, 8 isolates carried only single *gyrA* mutations with a CIP MIC of 1 μg/ml; 91 isolates carried single *gyrA* mutations and PMQR genes with CIP MICs ranging from 1 to ∼8 μg/ml; 22 isolates carried double *gyrA* and single *parC* mutations and exhibited CIP MICs of >16 μg/ml with and without PMQR genes; and 445 isolates only carried different PMQR genes, with CIP MICs ranging from 1 to ∼16 μg/ml.

Detailed analysis of the genome sequences of these *Salmonella* isolates revealed that various combinations of PMQR genes were present and that some of the resistance gene combinations appeared to be closely associated with specific serotypes. The *qnrS1-oqxAB*-*qnrS1* combination was the most common PMQR gene cluster and was found in 67% (375/566) of the ciprofloxacin-resistant *Salmonella* strains of various serotypes. The combination of *qnrS2* and *oqxAB–aac(6′)-Ib-cr* (112/566) was the second most common but was limited to *S*. Derby. The *qnrB6-qnrB4–aac(6′)-Ib-cr* (79/566) combination was commonly detectable in *S*. London and *S*. Kentucky. The *aac(6′)lb-cr–oqxAB* (80/566) combination was most commonly observed in *S*. Typhimurium (Table 3). In summary, the rapid increase in prevalence of ciprofloxacin resistance in *Salmonella* might be attributed to the following mechanisms: (i) horizontal transfer of PMQR gene-bearing mobile genetic elements among *Salmonella* strains and (ii) carriage of a single target mutation and acquisition of exogenous PMQR genes (14).

**TABLE 3:** Plasmids and TUs that harbored four major types of PMQR gene-bearing genetic elements in strains of different *Salmonella* serotypes^*a*^

Type	Reference	Size (bp)	CDS	% GC	Plasmid type	PMQR gene(s)	No.	Serotyping distribution	CS	Accession no.
1.1	p10kb	10,218	20	50.60	ColRNAI	*qnrS1*	269	Multiple serotypes	Y	CP025337
1.2	pSa1852-248kb	248,674	309	46.70	IncHI2A	*qnrS1*	28	*S*. Typhimurium, S. Derby, *S*. Corvallis, *S*. Weltevereden	C	MT513102
1.3	pSa21-28kb	27,772	41	51.10	IncX1	*qnrS1*	17	*S*. Agona (only)	Y	MH884649
1.4	pSa4-237kb	237,130	307	47.10	IncHI1B	*qnrS1*	13	*S*. Typhimurium, S. Give	C	MG874042
1.5	pSH01	64,226	85	53.50	IncFIA (HI1)	*qnrS1*	4	*S*. Meleagridis (only)	N	CP035381
1.6	TU-4kb	4,261	4	50	NA	*qnrS1*	21	*S*. Typhimurium, S. Rissen	N	—
1.7	TU-13kb	13,865	19	56.5	NA	*qnrS1*	4	*S*. Rissen	N	—
1.8	pSa27-186kb	186,308	186	46.60	IncHI2A	*qnrS1*, *oqxAB*	20	*S*. Goldcoast, *S*. Derby	C	MH884652
2	14-Sa79-chr-Cip	100,348	104	51.7	NA	*qnrS2–aac(6′)lb-cr–oqxAB*	112	*S*. Derby, *S*. Typhimurium, *S*. Corvallis	N	—
3.1	pSa76-CIP	104,666	130	54	IncFIB (K)	*qnrB6–aac(6′)lb-cr*	39	*S*. London (mainly), *S*. Typhimurium, *S*. Derby, *S*. Meleagridis	C	MG874044
3.2	pLA-64	64,226	85	53.0	IncHI2A	*qnrB6–aac(6′)lb-cr*	3	*S*. Typhimurium (only)	N	CP035381
3.3	TU_30kb	30,029	34	58.5	NA	*qnrB6–aac(6′)lb-cr*	27	*S*. Kentucky (only)	N	—
3.4	TU-44kb	44,092	50	53.6	NA	*qnrB6–aac(6′)lb-cr*	4	*S*. Corvallis (only)	N	—
3.5	Class I integron	22,977	25	51.8	NA	*qnrB4–aac(6′)lb-cr*	4	*S*. Thompson (only)	N	KY751925
4	pCFSA244-1	149,567	186	45.6	IncHI2A	*aac(6′)lb-cr–oqxAB*	81	*S*. Typhimurium (mainly)	N	CP033253

a CDS, coding sequences; GC, GC contents; CS, conjugation status; C, conjugative; Y, conjugative with the help of helper plasmid; N, nonconjugative. —, no accession number.

### Transmission of PMQR genetic mobile elements in ciprofloxacin-resistant *Salmonella*.

**FIG 2:**
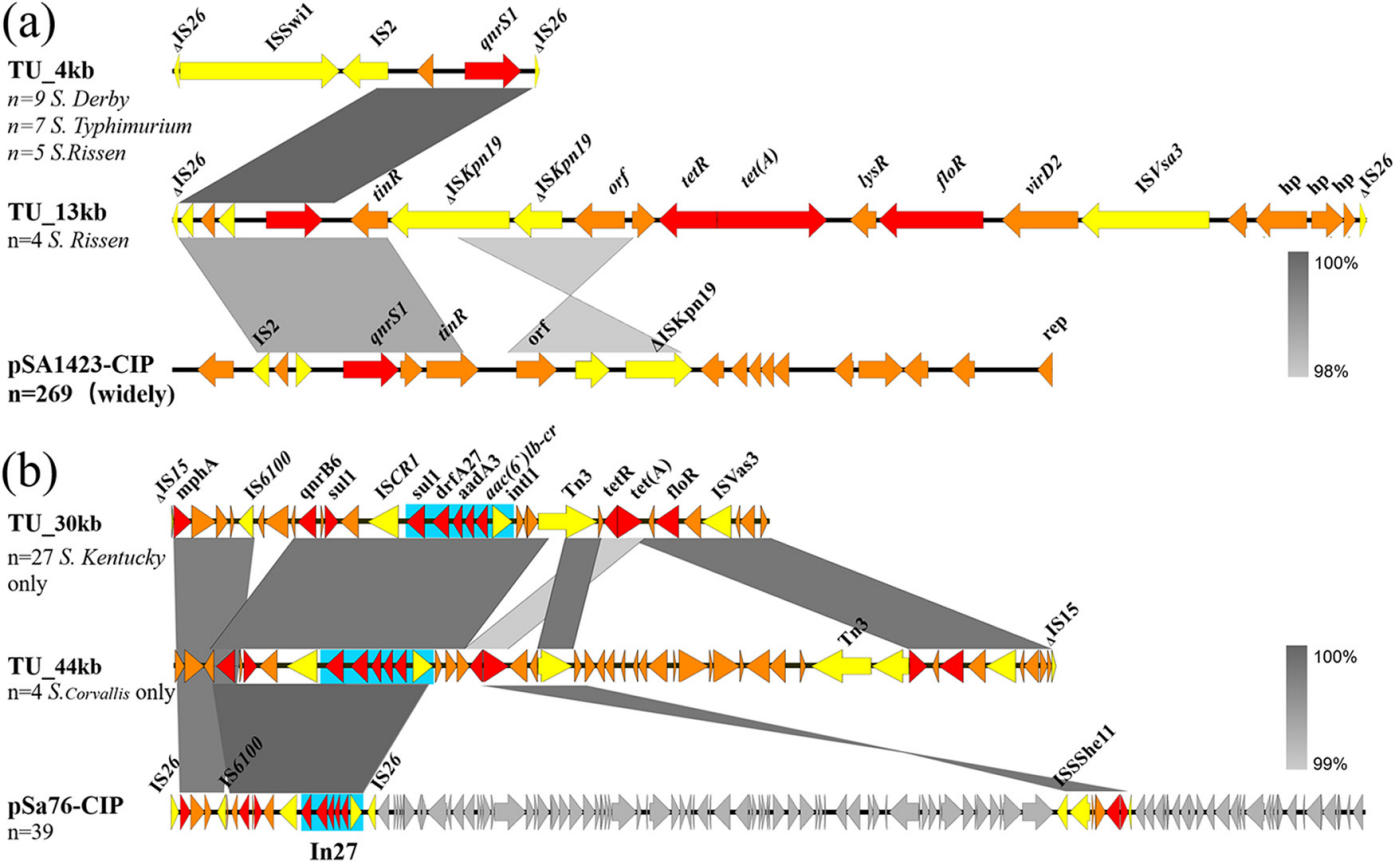
Structure alignment of different transposable units (TUs) carrying the *qnrS1–* or *qnrB6–aac(6′)lb-cr* genes, and comparison with plasmids with identical gene environments. (a) Alignment of TUs and plasmids carrying *qnrS1* using Easyfig. Representative TUs, including TU_4kb, TU_13kb, and p10-like plasmid pSA1423-CIP, were aligned. Key genetic loci in TUs and plasmids were labeled. TU sequence was generated from Illumina sequencing data. Red denotes the resistance genes, and yellow denotes ISs. (b) Alignment of TUs and plasmid carrying *qnrB6–aac(6′)lb-cr* by Easyfig. Plasmid pSa76-CIP was mainly prevalent in strains of the *S*. London serotype. It shares antibiotic resistance gene structure identical to that of two other transposable units, which could only be detected in *S*. Kentucky and *S*. Corvallis, respectively. Red denotes the resistance genes, yellow denotes ISs, and the blue region depicts the presence of an integron, In*27*.

It has been known that most of the PMQR genes in *Salmonella* are located in plasmids (19, 20). Therefore, the Cip^r^ *Salmonella* strains were subjected to screening of plasmids and gene cassettes harboring these PMQR mobile elements. A total of nine different types of plasmids, one chromosomal fragment, and five transposable units (TUs) were found to carry PMQR genes (Table 3). Six types of plasmids, namely, p10k-like plasmid (Fig. S1), pSA1892-248kb (Fig. S2a), pSa21-28kb (Fig. S2b), pSa4-237kb (Fig. S3a), pSH01 (Fig. S3b), and pSa27-186kb (Fig. S4a), and two types of TUs, including TU_4kb and TU_13kb, were found to harbor a *qnrS1-*bearing mobile element (Table 3, Fig. 2). Among these plasmids and TUs, plasmid p10k (GenBank accession no. CP025337), which belonged to the ColRNA1 plasmid type, exhibited a low-level ciprofloxacin resistance in *Salmonella* (13, 21–23). The second most common type of mobile element was those carrying the *qnrS2–aac(6′)lb-cr–oqxAB* resistance gene combination (Fig. S4b), which was often found in a chromosomal DNA fragment in *S*. Derby, *S*. Typhimurim, and *S*. Corvallis, with *S*. Derby being the most dominant (108/112). The third most common type was mobile elements carrying the *qnrB6-qnrB4–aac(6′)lb-cr* genes. Two kinds of plasmids, pSa76-CIP (Fig. S5a) and pLA_64kb (Fig. S5b), three kinds of TUs, TU_100kb, TU_30kb (Fig. S6a), and TU_44kb (Fig. S6b), and a class I integron (Fig. S7a) were involved in the transmission of this PMQR gene-bearing element. Most of these elements were located in pSa76-CIP, a conjugative plasmid. A few of them were located in nonconjugative plasmids, such as pLA-64, and TUs with unknown transmission potential. Unlike the plasmids and TUs, class I integron carrying the *qnrB4* gene was found in contigs of both 16,876 and 186,40 bp from *S*. Thompson, in which ISCR1 and genes encoding permeases (*sapA*, *sapB*, and *sapC*) and phage shock proteins (*pspA*, *pspB*, *pspC*, and *pspD*), as well as the AmpC-encoding *bla*_DHA-1_ gene, were also observed. This same region has been reported in different plasmids of various bacterial species (24). The fourth type of mobile element, carrying the *aac(6′)lb-cr–oqxAB* genes and located in a nonconjugative plasmid, pCFSA244-1, was mainly transmitted among *S*. Typhimurium isolates, with 73 isolates harboring such an element being detected (Fig. S7b). The transmission patterns of these PMQR genes in the major serotypes of ciprofloxacin-resistant *Salmonella* are described below (Table 3).

10.1128/mSystems.01234-20.4 FIG S1 Presence of p10kb-like plasmid in various serotypes of *Salmonella*. Alignment of 269 ciprofloxacin-resistant, nonconjugative ColRNAI-type plasmids from *Salmonella* using the BLAST Ring Image Generator (BRIG) is shown. (a) The sequence of p10kb in the outermost circle was used as a reference; key genetic loci are labeled. Resistance genes are highlighted in black, and yellow depicts insert sequence elements. (b) Dataset legend denotes distribution and serotypes belonging to these p10-like plasmids. Sequences were generated from the Illumina sequencing data. Download FIG S1, TIF file, 1.8 MB. Copyright © 2020 Chen et al. 2020 Chen et al. This content is distributed under the terms of the Creative Commons Attribution 4.0 International license.

10.1128/mSystems.01234-20.5 FIG S2 (a) Presence of IncHI2A, pSa1892-CIP-like plasmid in various serotypes of *Salmonella*. Alignment of ciprofloxacin-resistant conjugative IncHI2A-type plasmids from *Salmonella* using the BLAST Ring Image Generator (BRIG) is shown. The sequence of pSa1892-CIP in the outermost circle was used as a reference. Dataset legend denotes the prevalence and distribution of strains of various serotypes that harbor this plasmid; key genetic loci are labeled. Resistance genes are highlighted in black; yellow depicts insert sequence elements; *tra* genes responsible for conjugation are marked in green. Multidrug resistance genes flanked by a variety IS elements in the MDR region are labeled. Plasmid sequences were generated from the Illumina sequencing data. (b) Presence of IncX1, pSa21-CIP-like plasmid in various serotypes of *Salmonella*. Alignment of ciprofloxacin-resistant IncX1 -type plasmids from *Salmonella* using the BLAST Ring Image Generator (BRIG) is shown. The plasmid sequence of pSa21-CIP (accession number MH884651) in the outermost circle was used as a reference. Dataset legend depicts the prevalence and distribution of strains of various serotypes that harbor this plasmid; key genetic loci are labeled. Resistance genes are highlighted in black; yellow depicts insert sequence elements. Plasmid sequences were generated from the Illumina sequencing data. Download FIG S2, TIF file, 1.6 MB. Copyright © 2020 Chen et al. 2020 Chen et al. This content is distributed under the terms of the Creative Commons Attribution 4.0 International license.

10.1128/mSystems.01234-20.6 FIG S3 (a) Presence of IncHI1B, pSa4-CIP like plasmid in various serotypes of *Salmonella*. Alignment of ciprofloxacin-resistant conjugative IncHI1B-type plasmids from *Salmonella* using the BLAST Ring Image Generator (BRIG) is shown. The plasmid sequence of pSa4-CIP in the outermost circle was used as a reference; key genetic loci are labeled. Resistance genes are highlighted in black; yellow depicts insert sequence elements, and green denotes *tra* genes that encode conjugation functions. The double arrow denotes an unstable complex class I integron that preformed among these plasmids. Plasmid sequences were generated from the Illumina sequencing data. (b) Presence of IncR, pSH-01 like plasmid in various serotypes of *Salmonella*. Alignment of ciprofloxacin-resistant IncR-type plasmids from *Salmonella* using the BLAST Ring Image Generator (BRIG) is shown. The plasmid sequence of pSH-01 (accession number KY486279) in the outermost circle was used as a reference; key genetic loci are labeled. Resistance genes are highlighted in black color; yellow depicts insert sequence elements. Plasmid sequences were generated from the Illumina sequencing data. Download FIG S3, TIF file, 0.7 MB. Copyright © 2020 Chen et al. 2020 Chen et al. This content is distributed under the terms of the Creative Commons Attribution 4.0 International license.

10.1128/mSystems.01234-20.7 FIG S4 (a) Presence of IncHI2A, pSa27-CIP like plasmid in various serotypes of *Salmonella*. Alignment of ciprofloxacin-resistant IncHI2A-type plasmids from *Salmonella* using the BLAST Ring Image Generator (BRIG) is shown. The plasmid sequence of pSa27-CIP (accession number MH884652) in the outermost circle was used as a reference; key genetic loci are labeled. Resistance genes are highlighted in black; yellow depicts insert sequence elements. Plasmid sequences were generated from the Illumina sequencing data. (b) Presence of MDR chromosomal fragments Sa79-chr-cip in various serotypes of *Salmonella*. Alignment of chromosomal fragments Sa79-chr-cip from *Salmonella* using the BLAST Ring Image Generator (BRIG) is shown. The sequence of Sa79-chr-cip in the outermost circle was used as a reference to determine the prevalence of this MDR region among *Salmonella* strains. A total of 112 strains contained this MDR region, 108 of which belong to *S*. Derby. Key genetic loci are labeled. Resistance genes are highlighted in black, and yellow depicts insert sequence elements. Plasmid sequences were generated from the Illumina sequencing data. Download FIG S4, TIF file, 1.2 MB. Copyright © 2020 Chen et al. 2020 Chen et al. This content is distributed under the terms of the Creative Commons Attribution 4.0 International license.

10.1128/mSystems.01234-20.8 FIG S5 (a) Presence of IncFIB, pSa76-CIP-like plasmid in various serotypes of *Salmonella*. Alignment of ciprofloxacin-resistant conjugative IncFIB-type plasmids from *Salmonella* using the BLAST Ring Image Generator (BRIG) is shown. The sequence of pSa76-CIP in the outermost circle was used as a reference to determine the distribution pattern of this plasmid in this study; key genetic loci are labeled. Resistance genes are highlighted in black, yellow depicts insert sequence elements, and *tra* genes responsible for conjugation are highlighted in green. Plasmid sequences were generated from the Illumina sequencing data. (b) Presence of pLA-64-like plasmid in various serotypes of *Salmonella*. Alignment of pLA-64 (NCBI database accession number CP035381) with its best BLAST hit in this study. SA1307, SA1308, and SA1381, which belong to *S*. Typhimurium, were found to contain a pLA-64-like plasmid using the BLAST Ring Image Generator (BRIG). The pLA-64 plasmid was used as a reference (the outmost circle); key genetic loci are labeled. Plasmid sequences in this study were generated from the Illumina sequencing data. Download FIG S5, TIF file, 1.1 MB. Copyright © 2020 Chen et al. 2020 Chen et al. This content is distributed under the terms of the Creative Commons Attribution 4.0 International license.

10.1128/mSystems.01234-20.9 FIG S6 (a) Presence of TU-30kb in various serotypes of *Salmonella*. Alignment of TU-30kb carrying the *qnrB6–aac(6′)lb-cr* genes in *Salmonella* using the BLAST Ring Image Generator (BRIG) is shown. A total of 27 isolates of *S*. Kentucky recovered from 2015 to 2017 were analyzed; the sequence of TU_30kb from strain SA225 was used as a reference (the outmost circle); key genetic loci are labeled. TUs sequences were generated from the Illumina sequencing data. (b) Presence of TU_44kb in various serotypes of *Salmonella*. Alignment of TU_44kb carrying the *qnrB6–aac(6′)lb-cr* genes in *Salmonella* using the BLAST Ring Image Generator (BRIG) and Easyfig is shown. (Upper) Transport units in length with 44 kb from *Salmonella* isolates Sa292, SA574, SA585, and SA663 and plasmid p131681 (accession number MH114596) in the NCBI database were analyzed; p131681 exhibits 99% similarity with TU_44kb but only 48% coverage, suggesting that this type of TU has a novel structure. The sequence of TU_44kb from strain SA292 was used as a reference (the outermost circle); key genetic loci are labeled. TU sequences were generated from the Illumina sequencing data. (Lower) Structure alignment of TU_44kb carrying the *qnrB6–aac(6′)lb-cr* genes in *S*. Corvallis using Easyfig. Red, yellow, and purple denote the resistance, insert sequence elements, and hypothetical genes, respectively. Download FIG S6, TIF file, 0.8 MB. Copyright © 2020 Chen et al. 2020 Chen et al. This content is distributed under the terms of the Creative Commons Attribution 4.0 International license.

10.1128/mSystems.01234-20.10 FIG S7 (a) Presence of class I integron carrying the *qnrB4–aac(6′)lb-cr* genes in various serotypes of *Salmonella*. Alignment of class I integron carrying the *qnrB4–aac(6′)lb-cr* genes in *Salmonella* using the BLAST Ring Image Generator (BRIG) is shown. Key genetic loci are labeled. Resistance genes are highlighted in black, and yellow depicts insert sequence elements. (b) Presence of pCFSA244-1-like plasmid in various serotypes of *Salmonella*. Alignment of pCFSA244-1 carrying the *aac(6′)lb-cr–oqxAB* genes in *Salmonella* using the BLAST Ring Image Generator (BRIG) and Easyfig is shown. The sequence of pCFSA244-1 in the outermost circle was used as a reference; key genetic loci are labeled. Resistance genes are highlighted in black; yellow depicts insert sequence elements. The bar graph in the lower right corner depicts the prevalence of this plasmid in *Salmonella* strains of various serotypes; *S*. Typhimurium is the serotype that most commonly harbors this plasmid. Plasmid sequences were generated from the Illumina sequencing data. (c) Formation of a circular intermediate by the chromosomal fragment carrying *qnrS2–aac(6′)lb-cr–oqxAB* in *S*. Derby. (Left) Amplification of DNA fragment using primer set p1 (CTGGCGAAGACTCTCCGATG) and p2 (CGTCAGTCCATTGGCTTTGC), which suggested that the chromosomal fragment formed a circular intemediate. (Right) Illustration of the formation of circular intemediate by the chromosomal MDR fragment. Download FIG S7, TIF file, 0.8 MB. Copyright © 2020 Chen et al. 2020 Chen et al. This content is distributed under the terms of the Creative Commons Attribution 4.0 International license.

### Conjugative transmission of PMQR-bearing plasmids in *Salmonella*.

Transmission of PMQR genes in *Salmonella* can be mediated by conjugative plasmids or conjugative helper plasmids (17, 18). Unlike the pSa1852-248kb, pSa76-CIP, and pSa4-237kb plasmids that were self-conjugative, the conjugation process of pSa21-CIP and pSa27-CIP needs to be mediated by a conjugative helper plasmid. Accordingly, the IncI1 conjugative helper plasmids pSa21-HP and pSa27-HP could be detected in most (8/64) of the *Salmonella* strains that carried pSa76-CIP or pSa21-CIP (Table S1). Nonconjugative plasmids such as pLA-64 and pSH01 were reported in only a few isolates, with the exception of pCFSA244-1, which harbored the *aac(6′)lb-cr–oqxAB* element, which was detectable in 73 isolates of *S*. Typhimurium (Fig. S7b). The molecular mechanism underlying the conjugation of this plasmid was not fully understood, and more work is required to investigate how these plasmids are transmitted among *S*. Typhimurium strains. A chromosomal MDR fragment carrying the PMQR genes *qnrS2–aac(6′)lb-cr–oqxAB* was mainly detected in *S*. Derby, although it was occasionally detectable in other serotypes, such as *S*. Typhimurium (*n* = 3) and *S*. Corvallis (*n* = 1), suggesting that this chromosomal fragment can be transmitted among strains of different serotypes. Our data confirmed that this chromosomal fragment could form a circular intermediate, which may facilitate its transmission between different *Salmonella* strains (Fig. S7c).

**FIG 3:**
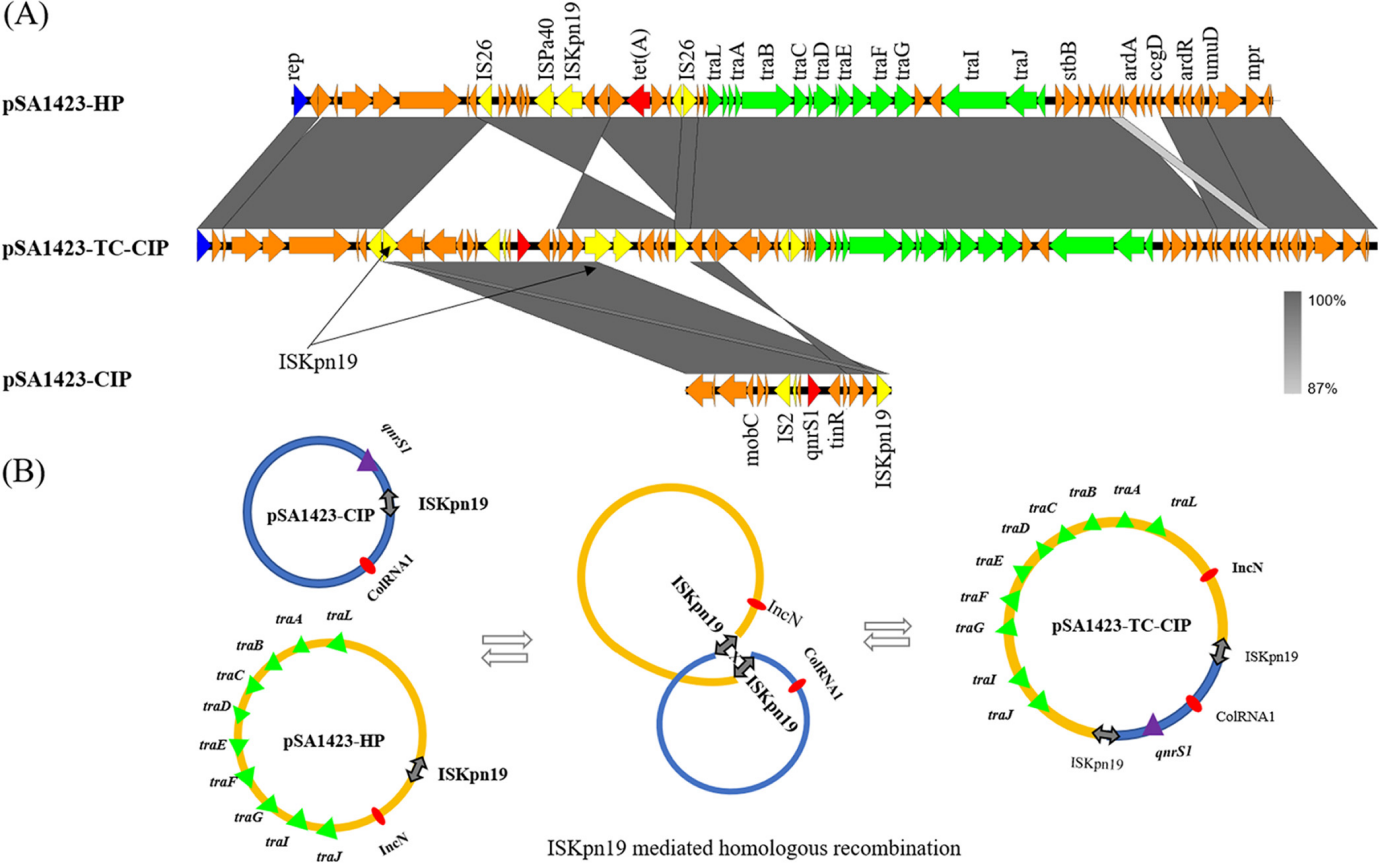
Mechanisms of plasmid recombination. (A) Structure alignment of three plasmids. Antimicrobial resistance genes, including *qnrS1* and *tet*(A) genes, are highlighted in red; yellow depicts IS elements, including IS*26*, IS*2*, and IS*Kpn19*; and the *tra* genes responsible for conjugation are shown by a green arrow. (B) Mechanism of plasmid fusion. Proposed IS element-mediated plasmid fusion through homologous recombination in *Salmonella* Sa1423. *pSa1423-HP* acted as the target plasmid, which attacked the hot spot in the donor plasmid, *pSa1423-CIP*, to facilitate plasmid fusion.

Importantly, the most predominant plasmid, p10k, was found to be widely distributed in multiple serotypes of *Salmonella* (Fig. S1, Table S2). This plasmid was first detected in 2014 and was found to be disseminated extensively among clinical *Salmonella* strains collected in 2015 to 2016. This phenomenon, which suggests that the transmission of p10k-like plasmids in *Salmonella* was not due to clonal transmission, is not consistent with the nonconjugative nature of this plasmid. To better understand the transmission mechanisms of this nonconjugative ColRNA1 plasmid, 50 randomly selected *Salmonella* strains carrying this plasmid were subjected to conjugation experiments using Escherichia coli J53 as the recipient strain. A total of 10 isolates could transfer the p10k-like plasmid to J53, suggesting that some currently unknown genetic mechanisms mediate the conjugation of p10k-like plasmid. One *Salmonella* strain, SA1423, and its transconjugant, SA1423-TC, were selected for complete plasmid sequencing by using both Illumina and Nanopore platforms. Our data showed that the p10k-like plasmid in SA1423 was a 10,218-bp plasmid that contained the *qnrS1* gene, with 50.6% GC content. It exhibited 99% identity at 99% coverage with plasmid p10k and was designated pSa1423-CIP. pSa1423-CIP was able to form a hybrid plasmid with a 49,279-bp conjugative plasmid and was designated pSa1423-HP, and it exhibited 71% identity at 99% coverage with the plasmid pVQS1 (GenBank accession no. JQ609357.1), carried by a *Salmonella* strain originating from a traveler in Germany, through homologous recombination at the IS*Kpn19* site (Fig. 3). Formation of the hybrid plasmid pSa1423-CIP-HP facilitated the transmission of pSa1423-CIP among different *Salmonella* isolates. Screening of pSa1423-HP in all *Salmonella* isolates identified 18 isolates (Table S3) that carried this helper plasmid, further confirming that this helper plasmid was involved in the transmission of p10k-like plasmids in *Salmonella* isolates.

10.1128/mSystems.01234-20.2 TABLE S2 Distribution and prevalence of plasmid pSA1423-CIP among strains of various *Salmonella* serotypes isolated during the period of 2013 to 2017. Download Table S2, DOCX file, 0.02 MB. Copyright © 2020 Chen et al. 2020 Chen et al. This content is distributed under the terms of the Creative Commons Attribution 4.0 International license.

10.1128/mSystems.01234-20.3 TABLE S3 Distribution and prevalence of helper plasmids among strains of various serotypes of *Salmonella* collected during the period of 2013 to 2017. Download Table S3, DOCX file, 0.02 MB. Copyright © 2020 Chen et al. 2020 Chen et al. This content is distributed under the terms of the Creative Commons Attribution 4.0 International license.

## DISCUSSION

The genetic features of foodborne *Salmonella* strains have changed significantly in recent years as ciprofloxacin-resistant *Salmonella* strains have become prevalent. Strains of some serotypes, such as *S*. Corvallis, *S*. Kentucky, and *S*. Stanley, that were highly resistant to ciprofloxacin have emerged. We observed that the mechanisms of ciprofloxacin resistance delineated in *Salmonella* strains have evolved significantly during this period. A major observation is that target gene mutations have become much less common in ciprofloxacin-resistant *Salmonella* strains. Currently, target gene mutations, in particular single mutation in the *gyrA* gene, may be detected in *S*. Typhimurium but is rarely seen in other serotypes of *Salmonella*. Double mutations in *gyrA* and single mutation in *parC* that mediated high-level resistance were seen only in *S*. Indiana, *S*. Kentucky, and *S*. Typhimurium strains but not in other serotypes (7, 25–27). Instead, PMQR genes, often in the form of multiple PMQR genes located in the chromosome and plasmids, now are commonly detectable in ciprofloxacin-resistant *Salmonella* strains of various serotypes. This shift in genetic location of ciprofloxacin resistance determinants from the chromosome to plasmids is responsible for the sharp increase in the prevalence of ciprofloxacin-resistant clinical *Salmonella* strains.

Mobile genetic elements carrying multiple PMQR genes are increasingly being detected in clinical *Salmonella* isolates and are responsible for the transmission of these ciprofloxacin resistance-encoding genes among such strains (16–18). The *qnrS1* gene was found to be harbored by multiple plasmids, with the p10k-like plasmids being the most dominant among a wide range of serotypes. The p10k-like plasmids were not conjugative but have become transmissible with the help of a helper plasmid, which could be lost upon being successfully transmitted to a new *Salmonella* strain. Our data showed that the widespread dissemination of p10k-like plasmids in *Salmonella* was closely associated with the emergence of the helper plasmid. Before 2015, only two *Salmonella* strains carrying p10k-like plasmids were detected, and no helper plasmid was found. In 2015, with the detection of pSA1423-HP, transmission of p10k in *Salmonella* accelerated, with as many as 119 isolates being found to carry this plasmid. The rate of detection of this plasmid continued to increase in 2016, with 133 isolates being found to carry this plasmid in that year. Meanwhile, the helper plasmid became detectable in a large number of isolates. In 2017, however, the helper plasmid was not detected in any strain, and the number of p10k-like plasmids in *Salmonella* was also found to have reduced dramatically. Other plasmids and TUs that carry the *qnrS1* gene have become dominant. This is an excellent example of dynamic evolution of ciprofloxacin resistance mechanisms in *Salmonella*, in which one dominant mechanism is not only gradually being replaced by another but also the transferrable nature of the new resistance mechanism resulted in a spike of the ciprofloxacin resistance rate of *Salmonella* to as high as 77% in 2017. In various serotypes of *Salmonella*, the *qnrS1* gene product was found to act together with other mechanisms to mediate the expression of the ciprofloxacin resistance phenotype. For example, in *S*. Typhimurium, it is often paired with a single *gyrA* mutation to cause ciprofloxacin resistance; in other serotypes of *Salmonella*, a combination of *qnrS1* and other PMQR genes located in chromosomal fragments or plasmids is often observed. In addition to being harbored by the p10k-like plasmid, the *qnrS1* gene can also be found in various conjugative and nonconjugative plasmids that are often serotype specific.

Another major mechanism of ciprofloxacin resistance involves the chromosomal fragment harboring the *qnrS2–aac(6′)lb-cr–oqxAB* elements. This chromosomal fragment, which was mainly detected in *S*. Derby, could produce a CIP MIC of ≥8 μg/ml. *S*. Derby isolates carrying this fragment were increasingly detected in recent years as a result of clonal spread. Our work showed that this chromosomal DNA fragment, flanked by IS*26*, was able to form a circular intermediate that facilitates the transmission of this DNA fragment between different *Salmonella* strains. The *qnrB6-qnrB4* and *aac(6′)lb-cr* genes were commonly detected in different plasmids and TUs that are uniquely present in different serotypes of *Salmonella*. One limitation of this study is that we are not able to depict the genetic structure of some TU-bearing plasmids and the mechanisms underlying the transmission of the pCFSA244-1 plasmid that contains the *aac(6′)lb-cr–oqxAB* elements in 80 isolates of *S*. Typhimurium. Since these isolates of *S*. Typhimurium are not derived from the same clone, clonal transmission of this plasmid should not be the sole mechanism of transmission of this ciprofloxacin resistance-encoding genetic fragment. Further studies are required to elucidate the mechanism of transmission of this nonconjugative plasmid.

Data from this study and others have suggested the underlying mechanisms of the rapid development of fluoroquinolone resistance in *Salmonella* spp. *Salmonella* strains used to be susceptible to fluoroquinolone antibiotics due to its low rate of development of mutations in their target genes, namely, double *gryA* mutations and single *parC* mutation, which cause high-level fluoroquinolone resistance (7, 8). Since their emergence around 2010, the PMQR genes have been found in the *Salmonella* chromosome or nonconjugative plasmid. The products of such genes produce low-level fluoroquinolone resistance when acting in combination with a single *gyrA* mutation, speeding up the development of fluoroquinolone resistance in *Salmonella* (15). The number and types of PMQR genes have increased rapidly in *Salmonella* due to transmission of these PMQR genes by conjugative plasmids and helper plasmids that could help conjugate a nonconjugative PMQR gene-coding plasmid to other *Salmonella* strains (16–18). The efficient transmission of PMQR genes in *Salmonella* enables one *Salmonella* strain to acquire multiple types of PMQR genes that could mediate the expression of low-level fluoroquinolone resistance in *Salmonella* without mutation in the target genes. The efficient transmission of PMQR genes resulted in rapid development of fluoroquinolone resistance in *Salmonella* in recent years. In conclusion, this study has provided comprehensive insight into the rapid evolution of ciprofloxacin resistance in *Salmonella* in the past 6 years. Continuous surveillance is warranted to depict the future trend of development of ciprofloxacin resistance in *Salmonella*.

## MATERIALS AND METHODS

### Bacterial isolates.

A total of 1,116 nonduplicate *Salmonella* isolates were collected from food samples during the period from 2012 to 2017 in Shenzhen, China. The species identities of these isolates were confirmed by detection of the *Salmonella*-specific gene *invA* and by using the matrix-assisted laser desorption ionization–time of flight mass spectrometry biotyper system (Bruker, Germany). Serotyping of the isolates then was performed according to the Kauffmann-White scheme by using a commercial antiserum (Difco, Detroit, MI), followed by testing of susceptibility to antimicrobial drugs according to CLSI guidelines (28). Ciprofloxacin-resistant isolates then were subjected to molecular screening for *qnrA*, *qnrB*, *qnrC*, *qnrD*, *qnrS*, *aac(6′)lb-cr*, and *oqxAB* as previously described (15). A total of 566 ciprofloxacin-resistant (Cip^r^) and 250 ciprofloxacin-sensitive (Cip^s^) isolates were selected for further investigation.

### Filter mating assay.

The transmission potential of PMQR genes was assessed by performing conjugation experiments, in which the filter-mating method was used as previously described (29). Transconjugants were selected on eosin methylene blue agar containing ciprofloxacin (0.5 mg/liter) and sodium azide (100 mg/liter). The transconjugants and their parental strains both were tested for their susceptibility to ciprofloxacin.

### Plasmid sequencing and analysis.

Conjugative plasmids were collected from the test strains and their corresponding transconjugants using the Qiagen Plasmid Midi kit (Qiagen, Valencia, CA). The Illumina platform and Nanopore MinION long-read sequencing platform were used to draft whole-plasmid maps. The Illumina paired-end libraries were prepared by using the NEBNext Ultra DNA library prep kit for Illumina (NEB) and then sequenced on an Illumina NextSeq 500 platform. *De novo* assemblies of MinION long reads and Illumina reads were performed with SPAdes 3.12.1 (30) and the CLC Genomics Workbench (CLC bio, Denmark), respectively. Long assembled contigs obtained from MinION long reads were used to align and join the contigs obtained from the Illumina assembly results. The completed plasmid sequence was annotated by the RAST tool (31) and the NCBI Prokaryotic Genome Annotation Pipeline (PGAP). All plasmids were sequenced using both Illumina and MinION long reads, and only high-quality data were used for further analysis. Screening and alignment of plasmids with similar structures were produced by BLAST Ring Image Generator (BRIG), version 0.95.22. (32).

### Whole-genome sequencing and genomic analysis.

A Pure-Link genomic DNA minikit (Invitrogen, USA) was used to extract DNA. DNA libraries were constructed by using the Nextera XT DNA sample preparation kit (Illumina, San Diego, CA, USA). Samples were multiplexed and sequenced on an Illumina Hiseq X for 300 cycles (250-bp paired end). Raw reads were trimmed and quality filtered using Trimmomatic v0.36 (33). Draft genomes were acquired by using SPAdes version 3.10.1 (34). Species identity and serotypes of the test strains were confirmed by SISTR (35). Whole-genome phylogenetic trees were created containing reference isolates for identification of *S*. Derby 14-Sa79. To detect PMQR genes and assess the distribution patterns of such genes in plasmids, the draft genome searches were conducted by BLAST (https://blast.ncbi.nlm.nih.gov/Blast.cgi), ResFinder (36), PlasmidFinder (37), and the CLC Genomics Workbench (CLC bio, Denmark) (Table 2). Integrons identified in the genomes were categorized according to INTEGRALL (http://integrall.bio.ua.pt).

### Phylogenetic analysis.

Trimmed and quality-filtered Illumina sequencing reads obtained from *Salmonella* strains were mapped to a ciprofloxacin-resistant *S*. Derby strain, 14-Sa79, which was sequenced using Nanopore and was confirmed to carry the *qnrS2–aac(6′)lb-cr–oqxAB* genes in its chromosome. Single-nucleotide polymorphisms (SNPs) were called using Snippy v3.1 with default settings (38), which used BWA-MEM v0.7.12 for short-read mapping. Snippy generates a core SNP alignment as well as a whole-genome SNP alignment. The whole-genome alignment was used to infer the ML phylogenies using Fasttree v2.1.10 with default parameters (39). The phylogenetic tree was visualized by iTOL version 3 (40). Eight hundred sixteen of the 1,116 *Salmonella* isolates, including 566 Cip^r^ and 250 Cip^s^ isolates, were included in this study. SISTR analysis, which identified 47 serotypes from 816 genomes, including 34 serotypes from 566 strains that exhibited the Cip^r^ phenotype, was also performed in the clustering analysis (41). Six serotypes of *Salmonella* that were predominantly prevalent among the foodborne strains and exhibited resistance to ciprofloxacin were also subjected to maximum likelihood phylogeny analysis.

### Data availability.

The sequencing data of the chromosome have been deposited in GenBank under BioProject ID PRJNA682289, and the sequences of the plasmids were deposited under the following accession numbers: pSA1852-248kb (MT513102), pSA1423-CIP (MK356559), pSA1423-HP (MK356560), pSA1423-TC-CIP (MK356561), and Sa79-chr (SGWG00000000).
