# Erratum for Chen et al., “Evolution of Ciprofloxacin Resistance-Encoding Genetic Elements in Salmonella”

**DOI:** 10.1128/msystems.00449-22

**Published:** 2022-05-31

**Authors:** Kaichao Chen, Chen Yang, Ning Dong, Miaomiao Xie, Lianwei Ye, Edward Wai Chi Chan, Sheng Chen

**Affiliations:** a Department of Infectious Diseases and Public Health, Jockey Club College of Veterinary Medicine and Life Sciences, City University of Hong Kong, Kowloon, Hong Kong; b State Key Laboratory of Chemical Biology and Drug Discovery, Department of Applied Biology and Chemical Technology, The Hong Kong Polytechnic University, Hung Hom, China; c Shenzhen Key Lab for Food Biological Safety Control, Food Safety and Technology Research Center, Hong Kong PolyU Shenzhen Research Institute, Shenzhen, China

## ERRATUM

Volume 5, no. 6, e01234-20, 2020, https://doi.org/10.1128/mSystems.01234-20. On page 1, an affiliation is missing from the first and last authors. The affiliations should appear as shown in this erratum.

On page 11, in the Acknowledgments section, “Basic Research Fund of Shenzhen City (20170410160041091)” should be revised to “Basic Research Fund of Shenzhen City (JCYJ20170818104557243).”

