# Supplementary material for: Evolution of Ciprofloxacin Resistance-Encoding Genetic Elements in Salmonella
Source: mSystems. 2020 Dec 22;5(6):e01234-20. doi: 10.1128/mSystems.01234-20 (PMC7762800; doi:10.1128/mSystems.01234-20)
Supplement: TABLE S1 [file mSystems.01234-20-st001.docx]

**Supplementary Table S1. Resistance rate, MIC_50_ and MIC_90_ of foodborne *Salmonella* isolates**

| **Antibiotics** | **Overall (n=1,116)** | | | **Strains resistant to Cip (n=566)** | | | **Strains resistant to CTX (n=140)** | | | **Strains resistant to AZI (n=58)** | | |
| --- | --- | --- | --- | --- | --- | --- | --- | --- | --- | --- | --- | --- |
|  | **Resistant rate (%)** | **MIC_50_ (μg/ml)** | **MIC_90_ (μg/ml)** | **Resistant rate (%)** | **MIC_50_ (μg/ml)** | **MIC_90_ (μg/ml)** | **Resistant rate (%)** | **MIC_50_ (μg/ml)** | **MIC_90_ (μg/ml)** | **Resistant rate (%)** | **MIC_50_ (μg/ml)** | **MIC_90_ (μg/ml)** |
|  |  |  |  |  |  |  |  |  |  |  |  |  |
| AMK | 2.86 | 4 | 8 | 4.77 | 4 | 8 | 5.00 | 4 | 8 | 18.97 | 4 | ＞128 |
| CTX | 12.51 | 0.12 | ≥16 | 13.60 | 0.12 | ≥16 | 100.00 | ≥16 | ≥16 | 25.86 | 0.12 | ＞16 |
| CRO | 12.51 | 0.12 | ≥16 | 13.60 | 0.12 | ≥16 | 100.00 | ≥16 | ≥16 | 25.86 | 0.12 | ＞16 |
| CIP | 50.58 | 1 | 8 | 100.00 | 2 | 16 | 55.00 | 1 | ≥16 | 81.03 | 2 | ＞16 |
| KAN | 25.56 | 4 | ≥128 | 41.17 | 16 | ≥128 | 42.14 | 8 | ≥128 | 41.38 | 32 | ＞128 |
| STR | 57.96 | 32 | ≥128 | 65.17 | 32 | ≥128 | 66.43 | ≥128 | ≥128 | 94.83 | ＞128 | ＞128 |
| TET | 75.60 | ≥32 | ≥32 | 90.11 | ≥32 | ≥32 | 92.14 | ≥32 | ≥32 | 94.83 | ＞32 | ＞32 |
| CHL | 51.47 | 32 | ≥64 | 80.21 | ≥64 | ≥64 | 66.43 | 64 | ≥64 | 89.66 | ＞64 | ＞64 |
| AZI | 5.18 | 1 | 16 | 8.30 | 1 | 16 | 10.71 | 1 | 32 | 100.00 | ＞32 | ＞32 |
| NAL | 42.45 | 16 | ≥64 | 60.07 | 32 | ≥64 | 46.43 | 32 | ≥64 | 44.83 | 16 | ＞64 |
| AMP | 58.09 | ≥64 | ≥64 | 67.14 | ≥64 | ≥64 | 95.00 | ≥64 | ≥64 | 87.93 | ＞64 | ＞64 |
| MRP | 0.00 | 0.03 | 0.12 | 0.00 | 0.03 | 0.12 | 0.00 | 0.06 | 0.12 | 0.00 | 0.03 | 0.12 |
| SXT | 59.61 | ≥32 | ≥32 | 70.67 | ≥32 | ≥32 | 71.43 | ≥32 | ≥32 | 91.38 | >32 | >32 |

Antibiotics and their breakpoints. AMK, amikacin, 64ug/ml; CTX, cefotaxime, 4ug/ml; CRO, ceftriaxone, 4ug/ml; CIP, ciprofloxacin, 1ug/ml; STR, streptomycin, 32ug/ml (FDA); KAN, kanamycin, 64ug/ml; TET, tetracycline,16ug/ml; CHL, chloramphenicol, 32ug/ml; AZI, Azithromycin, 32ug/ml: NAL, nalidixic acid, 32ug/ml; AMP, ampicillin, 32ug/ml; MRP: meropenem, 4ug/ml; SXT, sulfamethoxazole/trimethoprim, 4/76ug/ml;
