# Supplementary figures and images for: Evolution of Ciprofloxacin Resistance-Encoding Genetic Elements in Salmonella
Source: mSystems. 2020 Dec 22;5(6):e01234-20. doi: 10.1128/mSystems.01234-20 (PMC7762800; doi:10.1128/mSystems.01234-20)

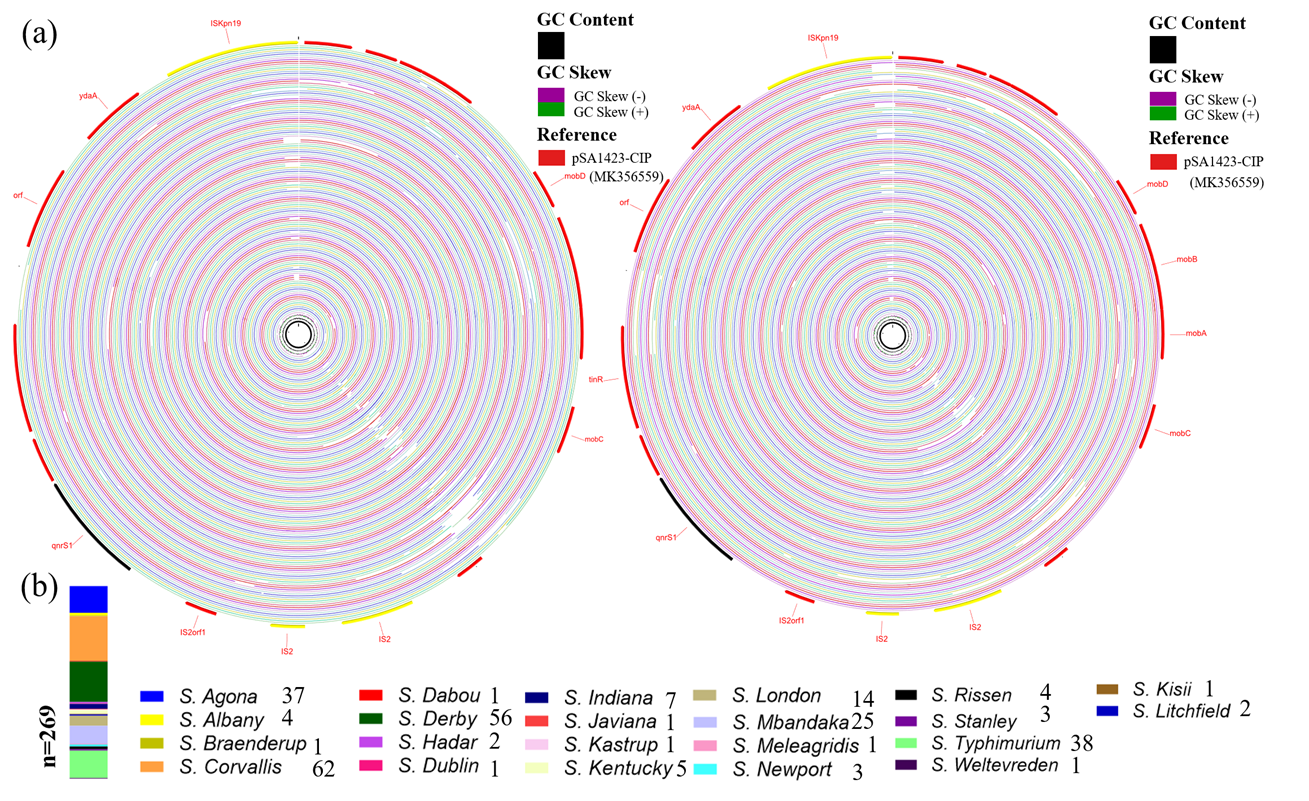

Supplement: FIG S1 [file mSystems.01234-20-sf001.tif]

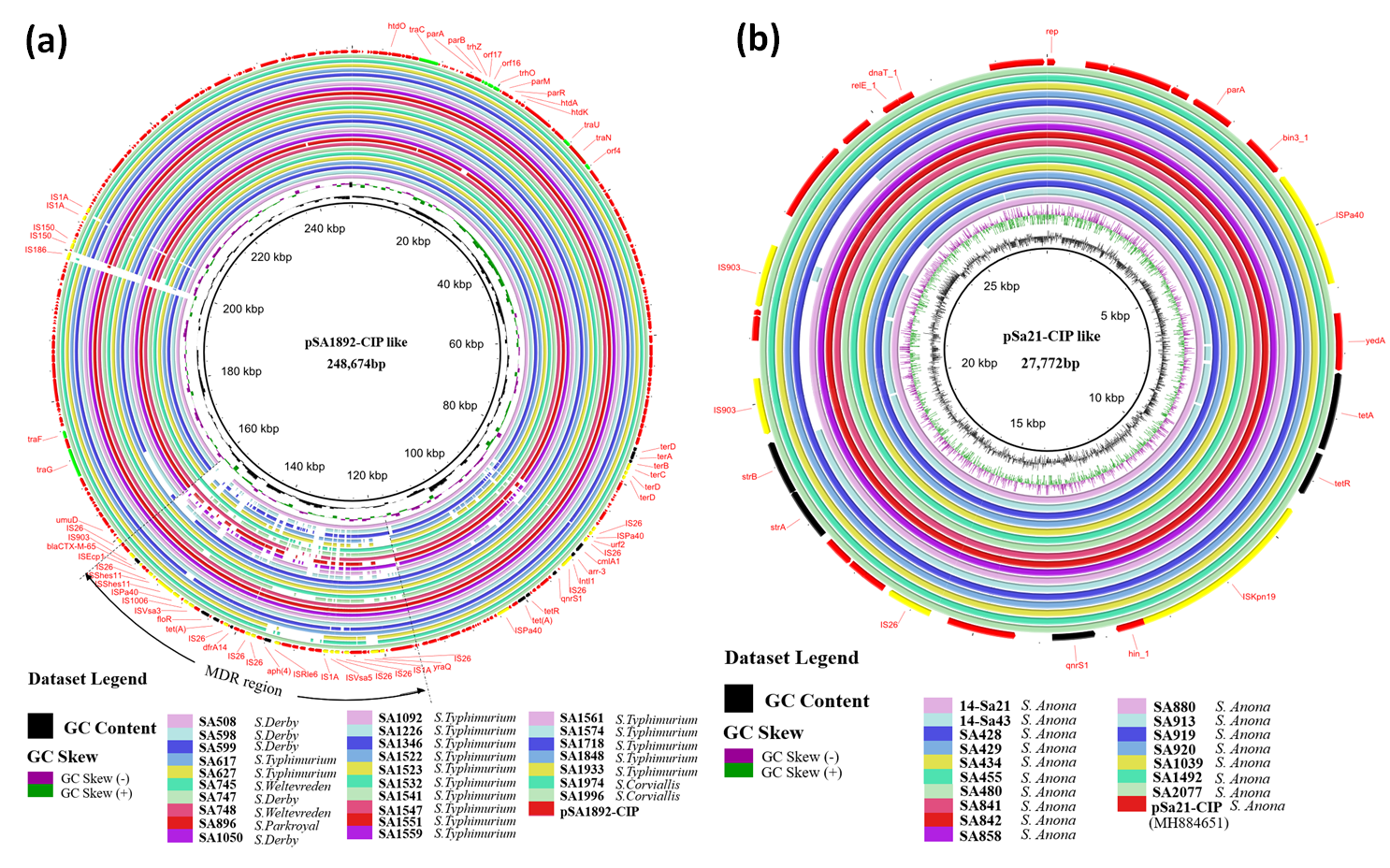

Supplement: FIG S2 [file mSystems.01234-20-sf002.tif]

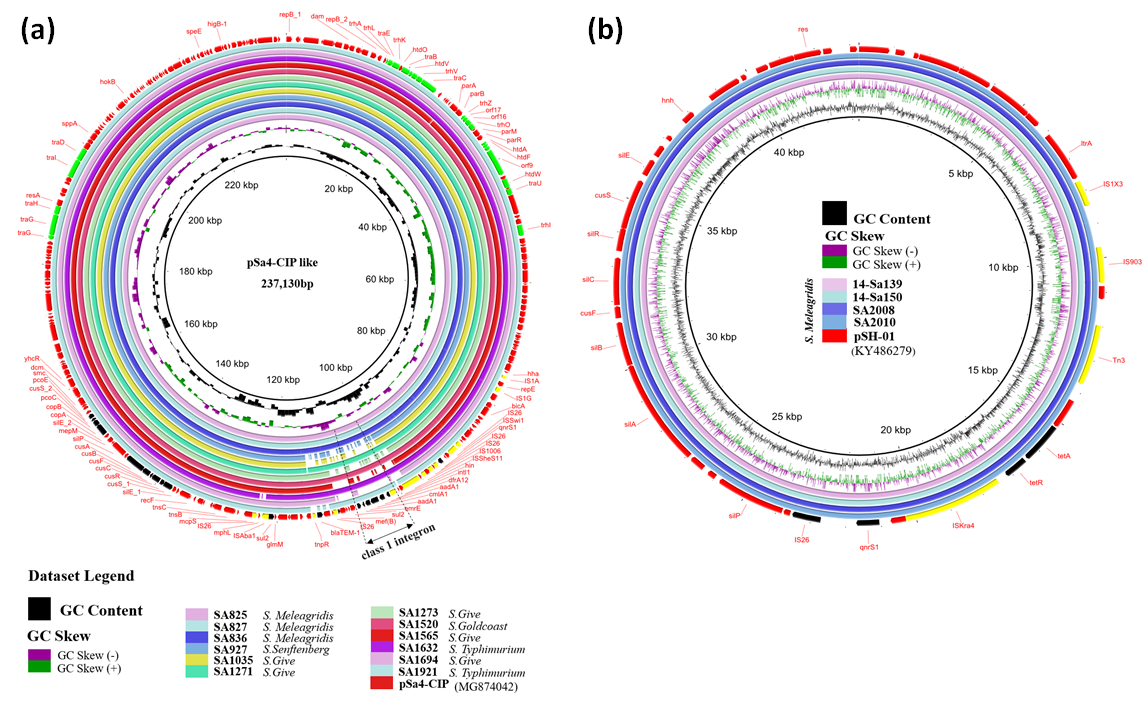

Supplement: FIG S3 [file mSystems.01234-20-sf003.tif]

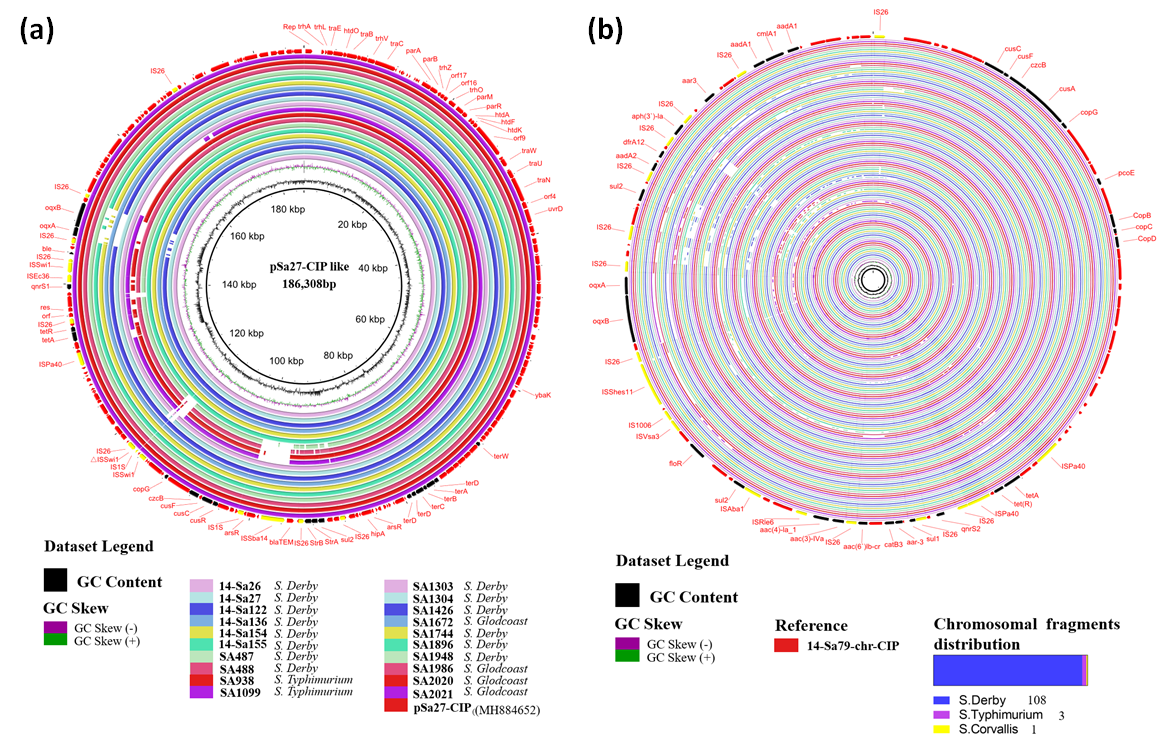

Supplement: FIG S4 [file mSystems.01234-20-sf004.tif]

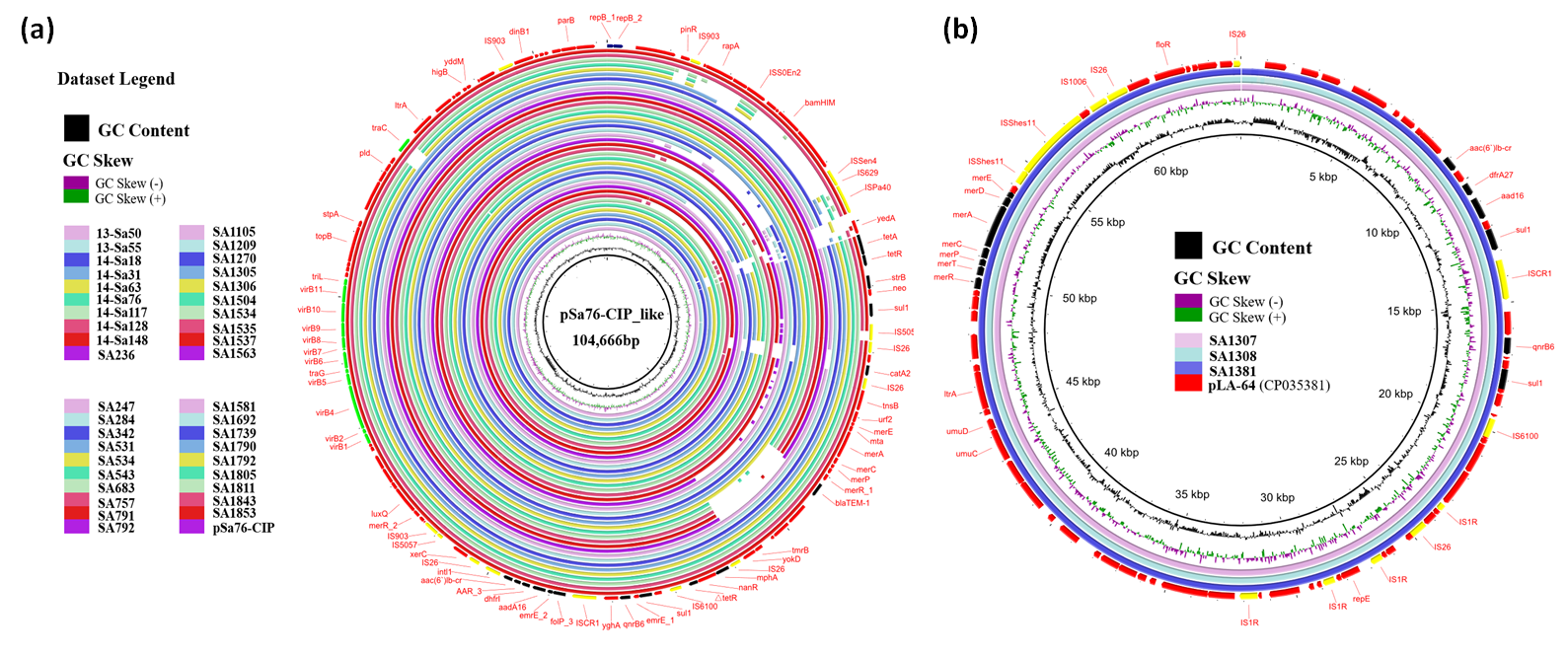

Supplement: FIG S5 [file mSystems.01234-20-sf005.tif]

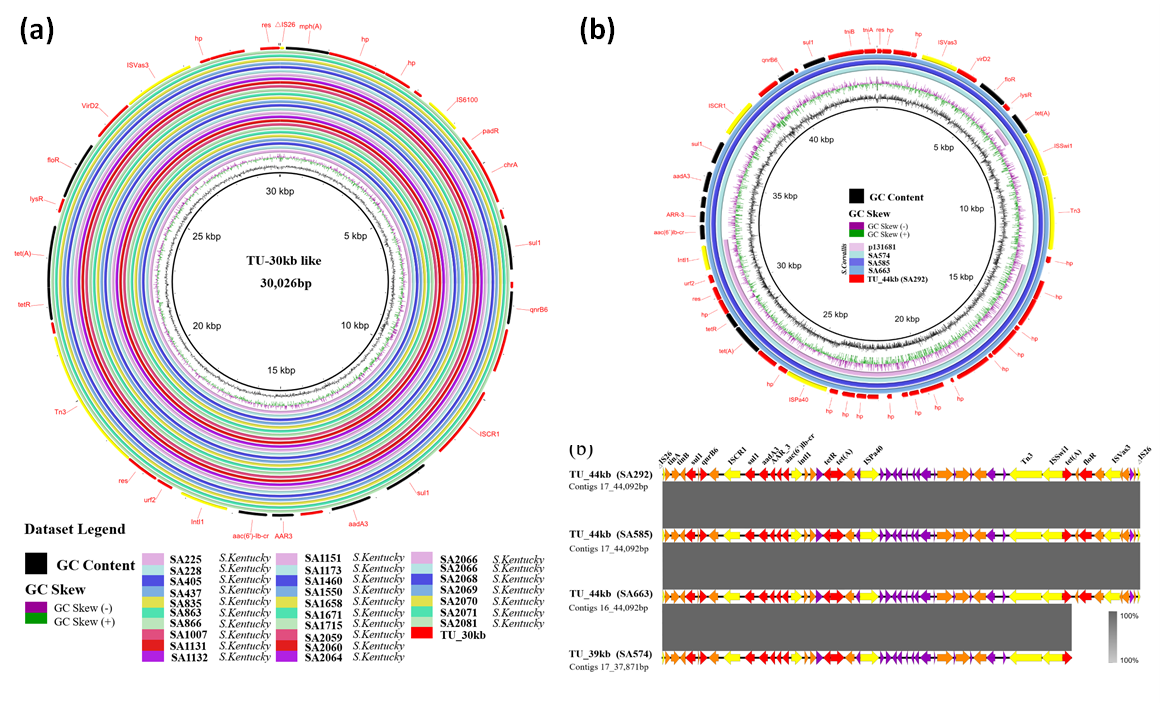

Supplement: FIG S6 [file mSystems.01234-20-sf006.tif]

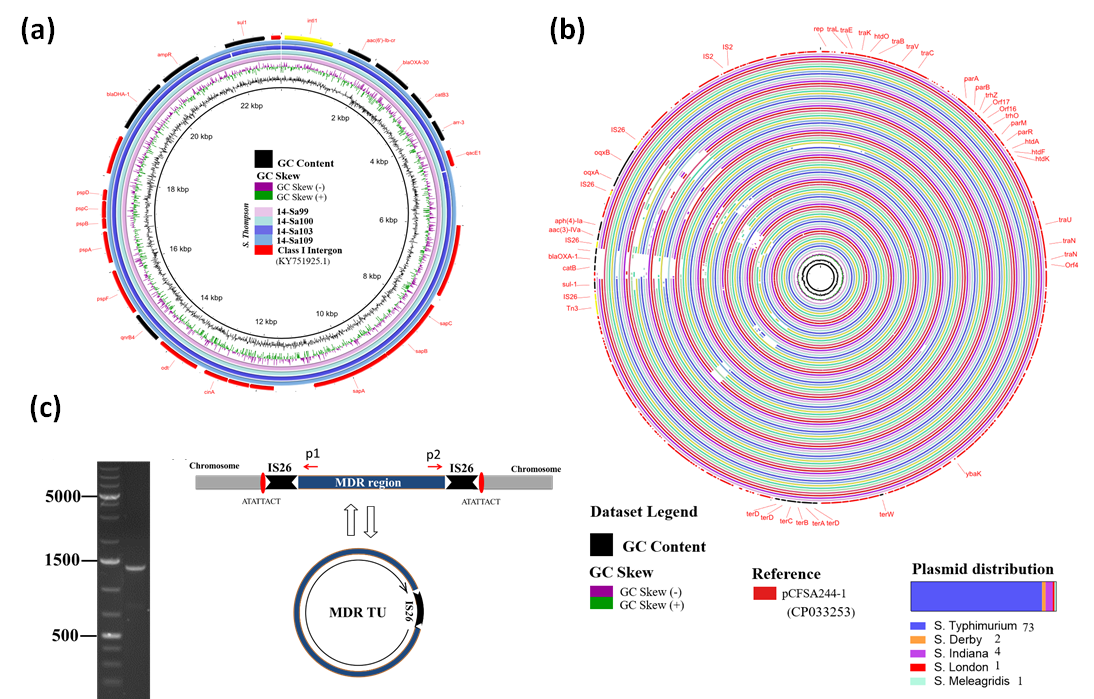

Supplement: FIG S7 [file mSystems.01234-20-sf007.tif]
