# Supplementary material for: Evolution of Ciprofloxacin Resistance-Encoding Genetic Elements in Salmonella
Source: mSystems. 2020 Dec 22;5(6):e01234-20. doi: 10.1128/mSystems.01234-20 (PMC7762800; doi:10.1128/mSystems.01234-20)
Supplement: TABLE S2 [file mSystems.01234-20-st002.docx]

**Supplementary Table S2. Distribution and prevalence of plasmid pSA1423-CIP among strains of various Salmonella serotypes isolated during the period 2013 to 2017.**

| **pSa1423-10kb** | **2013** | **2014** | **2015** | **2016** | **2017** | **Total** |
| --- | --- | --- | --- | --- | --- | --- |
| *S. Derby* | 0 | 1 | 30 | 25 | 1 | 56 |
| *S. Corvallis* | 0 | 0 | 19 | 35 | 6 | 60 |
| *S. Typhimurium* | 0 | 0 | 20 | 16 | 0 | 36 |
| *S. Agona* | 0 | 0 | 14 | 19 | 5 | 38 |
| *S. Mbandaka* | 0 | 0 | 8 | 6 | 0 | 14 |
| *S. London* | 0 | 1 | 8 | 6 | 0 | 15 |
| *S. Kentucky* | 0 | 0 | 6 | 11 | 2 | 19 |
| *S. Newport* | 0 | 0 | 3 | 0 | 0 | 3 |
| *S. Indiana* | 0 | 0 | 2 | 5 | 0 | 7 |
| *S. Weltevereden* | 0 | 0 | 1 | 0 | 0 | 1 |
| *S. Litchfield* | 0 | 0 | 2 | 0 | 0 | 2 |
| *S. Rissen* | 0 | 0 | 2 | 1 | 0 | 3 |
| *S. Goldcoast* | 0 | 0 | 1 | 0 | 0 | 1 |
| *S. Hadar* | 0 | 0 | 0 | 1 | 0 | 1 |
| *S. Dabou* | 0 | 0 | 1 | 0 | 0 | 1 |
| *S. Kisii* | 0 | 0 | 1 | 0 | 0 | 1 |
| *S. Meleagridis* | 0 | 0 | 0 | 1 | 0 | 1 |
| *S. Stanley* | 0 | 0 | 0 | 1 | 1 | 2 |
| *S. Albany* | 0 | 0 | 0 | 4 | 0 | 4 |
| *S. Kastrup* | 0 | 0 | 1 | 0 | 0 | 1 |
| *S. Doblin* | 0 | 0 | 0 | 1 | 0 | 1 |
| *S. Javiana* | 0 | 0 | 0 | 1 | 0 | 1 |
| total | 0 | 2 | 119 | 133 | 15 | 269 |
