# Supplementary material for: Evolution of Ciprofloxacin Resistance-Encoding Genetic Elements in Salmonella
Source: mSystems. 2020 Dec 22;5(6):e01234-20. doi: 10.1128/mSystems.01234-20 (PMC7762800; doi:10.1128/mSystems.01234-20)
Supplement: TABLE S3 [file mSystems.01234-20-st003.docx]

**Supplementary** **Table S3. Distribution and prevalence of helper plasmids among strains of various serotypes of *Salmonella* collected during the period 2013 to 2017.**

| **Plasmids** | **Plasmid types** | **Plasmid distribution** | **Year of isolation** | | | | | **Total** |
| --- | --- | --- | --- | --- | --- | --- | --- | --- |
|  |  |  | **2013** | **2014** | **2015** | **2016** | **2017** |  |
| pSa21-HP & pSa27-HP | IncI1 | *S. Derby*(mainly)*, S. Agona, S., London, S. Rissen*  *S. typhimurium, S. Enteritidis, S. Corvallis etc.* | 22 | 12 | 9 | 12 | 9 | 64 |
| pSA1423-HP | IncN | *S. Agona (n=17), S. Corvallis (n=1)* | 0 | 0 | 5 | 13 | 0 | 18 |
